# Rural–urban differences in health care access for postpartum parent and infant dyads

**DOI:** 10.1111/jrh.70062

**Published:** 2025-08-03

**Authors:** Sara C. Handley, Julia D. Interrante, Emily F. Gregory, Katy B. Kozhimannil

**Affiliations:** ^1^ Department of Pediatrics Perelman School of Medicine at the University of Pennsylvania Philadelphia Pennsylvania USA; ^2^ Leonard Davis Institute of Health Economics The University of Pennsylvania Philadelphia Pennsylvania USA; ^3^ Division of Health Policy & Management University of Minnesota School of Public Health Minneapolis Minnesota USA

**Keywords:** access to care, health services research, medical care, utilization of health services

## Abstract

**Purpose:**

To examine differences in perinatal health between rural and urban postpartum parents and infants and within postpartum parent–infant dyads.

**Methods:**

Cross‐sectional analysis of the National Health Interview Survey (NHIS) data. Accounting for the complex survey design, we calculated weighted proportions of measures of self‐rated health, health care utilization, and barriers to care and used chi‐squared tests to assess rural–urban differences between postpartum parents and between infants, and repeated measures to test postpartum parent–infant differences within households in rural and urban counties.

**Findings:**

The study included 2019 rural postpartum parents, 2191 rural infants, 12,112 urban postpartum parents and 13,088 urban infants. Compared to urban postpartum parents, those living in rural areas were less likely to see an obstetrician–gynecologist (*p* = 0.002) had more emergency department (ED) visits (*p* = 0.030), reported more hospitalizations (*p* = 0.041), more frequently experienced uninsurance (*p* = 0.006), and lost Medicaid coverage after pregnancy (*p* = 0.006). While a higher proportion of urban infants were hospitalized than their rural counterparts (*p* = 0.019), other measures were similar. Accounting for dyad correlations, compared to infants, postpartum parents generally reported worse health (fair or poor self‐rated health), and were more likely to experience ED visits, hospitalizations, loss of health care coverage, and barriers to care.

**Conclusions:**

Rural postpartum parents experience worse health than their urban counterparts and compared to their infants. Rural–urban differences in access were less common among infants, thus leveraging infant care systems for services to both the infant and postpartum parent may improve household health in all communities.

## INTRODUCTION

Maternal and infant mortality during the postpartum period (the year following birth) is a persistent problem in the United States.^1^, ^2^ Further, maternal–infant dyads residing in rural areas experience higher rates of adverse outcomes, including mortality.^3^, ^4^ Rural–urban differences exist in baseline health status, utilization of routine and acute health care services, as well as unique structural barriers to care.^5^, ^6^, ^7^, ^8^ These differences often reflect “structural urbanism.”^8^ Structural urbanism biases health care toward large population centers via market forces associated with higher patient volume and prioritization of public health efforts in more densely populated areas.^8^ For maternal–infant dyads living in rural communities, structural urbanism manifests as workforce shortages and limited availability of essential services, such as primary pediatric care, midwifery care, hospital‐based obstetric services, and routine neonatal care after birth.^9^, ^10^, ^11^, ^12^, ^13^ This is in addition to rural‐specific barriers related to transportation and longer travel distances to reach essential health care services.^14^ These differences in rural health care availability and access likely compound challenges in obtaining postpartum care for maternal–infant dyads, who each have specific, time‐sensitive, and important health care needs.

Establishing health care during the postpartum period is important to the future well‐being of the parent–infant dyad.^15^, ^16^, ^17^ This period represents a critical transitional phase, marked by unique physical, psychological, and social vulnerabilities for both postpartum parents and infants.^15^, ^16^, ^17^ The persistent challenges in addressing preventable postpartum pregnancy‐related deaths and sudden unexpected infant death illustrate these unique vulnerabilities.^1^, ^18^ These adverse outcomes have motivated national organizations to redefine and optimize postpartum care and create targeted infant death prevention efforts.^16^, ^19^ Such efforts recognize that the postpartum period is an ongoing, individualized process encompassing physical recovery from birth, preventative and chronic disease management, contraception and birth spacing, mental health, and infant feeding and care.^16^ Ongoing, longitudinal care is a central concept in infant care, with 7 visits recommended in the first year to monitor growth, assess development, and provide preventative care.^17^ Despite the importance of care during the postpartum period, there are differences between postpartum parents and infants in the percentage that receive this care. On average 72% of postpartum parents attend a postpartum visit (though estimates range from 25% to 96%), while more than 80% of infants have a health visit within the first year.^20^, ^21^, ^22^ These data vary markedly by geography, income, and insurance status.^20^, ^21^, ^22^ Prioritization of infant health over postpartum parent health has been documented among parents of preterm infants.^23^, ^24^ As infants are born at earlier gestational ages, a decreasing proportion of parents receive postpartum care.^23^ Parents of preterm infants often pursue care for their infant more actively than they engage in preventative obstetric care for themselves.^24^ Postpartum parents of both ill and well infants are faced with the challenge of managing concurrent health and health care needs for both their infants and themselves. The concurrent and potentially competing needs can strain personal and family resources, including time, social support, technology access, and finances.

Throughout pregnancy, birth, and the postpartum period, the health and well‐being of parents and infants are interrelated. For example, pregnant people with high‐risk conditions (e.g., preeclampsia, psychiatric conditions, substance use disorders) are more likely to give birth to high‐risk infants who are born preterm or require intensive care after birth.^25^, ^26^, ^27^ Within these dyads, both the parent and infant require additional care and support in the postpartum period.^25^, ^26^, ^27^ The interrelated health of the parent–infant dyad after childbirth is not limited to higher risk dyads. It includes postpartum parent mental health, infant bonding, and breastfeeding, which has health benefits for both members of the dyad.^28^, ^29^ Navigating the health care needs and challenges of the postpartum period may be distinct for parents and infants who live in rural and urban areas.^30^, ^31^ A better understanding of the challenges faced by rural and urban postpartum parents and their infants, both individually and within the dyad, has the potential to inform health care systems, health care delivery, and postpartum‐specific policies.

Our objective was to examine differences in self‐rated health, health care utilization, and barriers to care between rural and urban postpartum parents and infants and within postpartum parent–infant dyads. We hypothesized that dyads residing in rural areas would report less care utilization and more barriers to care compared to those residing in urban areas and that within the dyad, infant care would be prioritized over care for the postpartum parent.

## METHODS

### Study design and population

This is a pooled cross‐sectional cohort study using data from the National Health Interview Survey (NHIS) collected 2006–2018.^32^ The NHIS data captures a nationally representative, civilian, non‐institutionalized population via an in‐person interview survey and has been continuously collected since 1957. A full description of the survey, survey item measurement, and evaluation details from the Collaborating Center for Questionnaire Design and Evaluation Research are publicly available.^32^, ^33^ Data collection for the NHIS was approved by the National Center for Health Statistics (NCHS) Research Ethics Review Board (ERB). Analysis of de‐identified data from the survey is exempt from the federal regulations for the protection of human research participants. Analysis of restricted data through the NCHS Research Data Center is also approved by the NCHS ERB. The restricted dataset was accessed through the Integrated Public Use Microdata Health Surveys^34^ at the University and the Research Data Center. This study was reviewed and designated exempt by the senior author's Institutional Review Board.

We included non‐pregnant female‐identifying adults of reproductive age (18–49 years old) who had infants ≤1 year old. Identification of postpartum parents was dependent on an item indicating that the respondent had a live birth in the last 12 months. Information about the sample child (infant <1 year old) is obtained from the sample child respondent, an adult residing in the household who is knowledgeable about the child's health (householder), which may be the postpartum parent or another adult household member. Given that this is a pooled cross‐sectional study, some postpartum parents are likely reporting visits that occurred during the third trimester of pregnancy and not all infants would have had time to complete the 7 recommended visits during the first year after birth.

### Outcomes and variables

The outcomes of interest were various measures of health status, health care utilization, and barriers to health care for postpartum parents and infants. With respect to health status, we used self‐rated health for the postpartum parent and infant rated separately on a 5‐point scale: excellent, very good, good, fair, poor.

Health care utilization was examined for the postpartum parent and infant using variables reporting (1) the location where usual care was received (clinic/health center, doctor's office, hospital emergency department [ED]); (2) the total number of office visits in the prior year (referred to as “office visits”); (3) visits with a specific type of clinician for the respective members of the dyad; a visit with an obstetrician–gynecologist, a visit with a nurse practitioner (NP), physician assistant (PA), or midwife, and a well child check (WCC); (4) the number of ED visits in the prior year (referred to as “ED visits”); and (5) number of hospitalizations (excluding the hospitalization for childbirth).

Barriers to accessing health care were examined under two broad categories. First, health insurance coverage, including (1) experiencing any time without coverage in the prior year (referred to as “uninsurance”); (2) coverage stopping after pregnancy; and (3) changes in the usual place of care based on insurance coverage. The second category included reasons for delays in medical care in the last year.

The exposure of interest was rural or urban residence. In these data, location of residence is a restricted geographic variable requested from the US Census Bureau's Research Data Center in order to merge patient files with this key geographic measure for analysis. Classification of rural or urban was determined using the county of residence per the NCHS Urban‐Rural Classification Scheme based on the US Office of Management and Budget definitions of metropolitan and non‐metropolitan county status.

We examined a variety of household, postpartum parent, and infant sociodemographic characteristics that were self‐reported by survey respondents. Household characteristics included Census region (Midwest, Northeast, South, West), being below the federal poverty threshold, food security scale (secure, low, very low), and number of children in the household (1, 2, ≥3). Postpartum parent characteristics included age, race and ethnicity (Hispanic, Non‐Hispanic American Indian/Alaskan Native, Non‐Hispanic Asian, Non‐Hispanic Black, Non‐Hispanic White, Other/Multiple), preferred language (English, Spanish), marital status, education status, employment status, primary payer (private, public, uninsured), and reported problems paying medical bills in the past year. Infant characteristics included birth weight, race and ethnicity, and primary payer. These variables, including race and ethnicity, were selected to reflect the structural barriers and resources within the households of postpartum parents and infants that may influence their health and access to health care.^35^, ^36^

### Statistical analysis

To assess self‐reported health measures and health care utilization, the survey data were pooled and weighted in accordance with NHIS recommendations (including variables for strata and primary sampling units).^37^ This approach accounts for complex survey design, nonresponse bias, and multiple survey cycles. We used survey‐weighted analyses to calculate proportions and 95% confidence intervals (CI) and used chi‐squared and two‐sample *t*‐tests to assess bivariate differences between rural and urban postpartum parents and infants. To examine within dyad differences, the study population was subset to postpartum parents and infants from the same household. As recommended by NCHS,^38^ we tested for bivariate differences between postpartum parents and their infant by fitting a repeated measures regression model at the household level that accounted for the relevant outcome measure of the other dyadic member. We calculated predicted percentage point differences within dyads, among rural and urban residents. Analyses were conducted using Stata version 18.0.

## RESULTS

The study population included 2019 rural postpartum parent respondents and 2191 rural infants and 12,112 urban postpartum parent respondents and 13,088 urban infants. Compared to urban dyads, rural dyads were more likely to be living below the federal poverty threshold (rural 34.3% vs. urban 24.4%) and have more children in the household (≥3 children rural 35.3% vs. urban 31.4%) (Table 1). Compared to urban postpartum parents, rural postpartum parents were younger (mean age 27.1 years), more likely to be Non‐Hispanic White (76.1%), speak English (95.4%), be unmarried (63.4%), less likely to have attained a college degree (17.1%), more likely to be publicly insured (31.0%) or uninsured (22.4%), and more likely to report problems paying medical bills (26.0%) (Table 1). While there was no rural–urban difference for infant birth weight, rural infants were also more likely to be Non‐Hispanic White (72.6%) and publicly insured (54.2%) or uninsured (6.2%) (Table 1).

**TABLE 1:** Household, postpartum parent, and infant characteristics stratified by rural–urban residence.

Characteristics	Rural weighted % (95% CI)	Urban weighted % (95% CI)	*p* Value
**Household, unweighted *n***	**(*n* = 2191)**	**(*n* = 13,088)**	
Census region			0.000
Midwest	37.3 (32.7–42.1)	20.6 (19.5–21.9)	
Northeast	7.8 (5.7–10.7)	16.8 (15.8–17.8)	
South	41.0 (36.1–46.2)	37.8 (36.4–39.3)	
West	13.9 (10.8–17.6)	24.8 (23.5–26.1)	
Only one adult in the household	11.2 (9.6–13.1)	10.2 (9.6–10.9)	0.295
Below federal poverty threshold	34.3 (31.7–36.1)	24.4 (23.4–25.5)	<0.001
Food security scale			0.408
Secure	86.7 (84.7–88.4)	88.2 (87.3–89.1)	
Low	8.8 (7.3–10.7)	7.4 (6.8–8.1)	
Very low	4.4 (3.2–5.9)	4.3 (3.8–4.9)	
Number of children in household			<0.001
One	29.5 (27.6–31.5)	34.4 (33.4–35.4)	
Two	35.2 (32.7–37.8)	34.2 (33.1–35.3)	
Three or more	35.3 (32.8–37.8)	31.4 (30.3–32.6)	
**Postpartum parent, unweighted *n***	**(*n* = 2019)**	**(*n* = 12,112)**	
Age (mean)	27.1 (26.8–27.5)	29.2 (29.1–29.3)	<0.001
Race and ethnicity			<0.001
Hispanic	10.8 (7.3–14.2)	23.6 (22.6–24.7)	
Non‐Hispanic American Indian/Alaska Native	2.6 (1.2–3.9)	0.6 (0.4–0.8)	
Non‐Hispanic Asian	1.0 (0.5–1.5)	3.9 (3.5–4.3)	
Non‐Hispanic Black	8.6 (6.9–10.2)	14.4 (13.5–15.3)	
Non‐Hispanic White	76.1 (72.3–80.0)	54.5 (53.2–55.9)	
Other/multiple	1.0 (0.4–1.7)	2.9 (2.6–3.2)	
Language			<0.001
English	95.4 (92.6–97.2)	89.5 (88.7–90.2)	
Spanish	4.5 (2.7–7.4)	9.9 (9.2–10.7)	
Marital status			<0.001
Married	63.4 (60.7–66.1)	68.5 (67.4–69.6)	
Divorced/separated/widowed	7.0 (5.8–8.1)	5.4 (4.9–5.9)	
Never married	29.3 (26.7–31.8)	25.9 (24.8–27.0)	
Educational attainment			<0.001
<High school	16.9 (14.6–19.2)	14.3 (13.5–15.1)	
High school degree	30.6 (28.1–33.2)	21.8 (20.9–22.8)	
Some college	34.5 (31.9–37.1)	28.5 (27.6–29.5)	
College degree or more	17.1 (15.2–19.1)	34.3 (33.0–35.5)	
Employment status			0.088
Employed (full or part time)	51.0 (48.0–54.0)	51.0 (49.9–52.2)	
Unemployed	5.1 (4.1–6.1)	5.2 (4.8–5.7)	
Not in labor force	43.7 (40.9–46.6)	42.9 (41.7–44.0)	
Primary payer			<0.001
Private	46.1 (43.3–48.9)	59.3 (58.1–60.6)	
Public	30.9 (27.9–33.9)	24.0 (22.9–25.1)	
Uninsured	22.4 (19.9–24.9)	16.2 (15.3–17.0)	
Problems paying or unable to pay medical bills, past 12 months	26.0 (22.9–29.1)	20.3 (19.2–21.4)	<0.001
**Infant, unweighted *n***	**(*n* = 2191)**	**(*n* = 13,088)**	
Birth weight, grams (mean)	3293 (3247–3339)	3278 (3260–3296)	0.553
Race and ethnicity			<0.001
Hispanic	13.9 (10.6–18.1)	27.3 (26.2–28.4)	
Non‐Hispanic American Indian/Alaska Native	2.5 (1.4–4.2)	0.6 (0.4–0.9)	
Non‐Hispanic Asian	0.7 (0.4–1.2)	3.1 (2.8–3.4)	
Non‐Hispanic Black	9.3 (7.5–11.4)	15.2 (14.3–16.1)	
Non‐Hispanic White	72.6 (68.4–76.4)	50.5 (49.2–51.9)	
Other/multiple	1.1 (0.6–2.1)	3.3 (3.0–3.7)	
Primary payer			<0.001
Private	39.1 (36.4–41.9)	53.0 (51.8–54.3)	
Public	54.2 (51.4–56.9)	43.2 (41.9–44.4)	
Uninsured	6.2 (5.0–7.8)	3.3 (3.0–3.7)	

*Note*: Data are weighted to account for complex survey design, nonresponse bias, and multiple survey cycles.

Abbreviation: CI, confidence Interval.

### Self‐rated health

**FIGURE 1:**
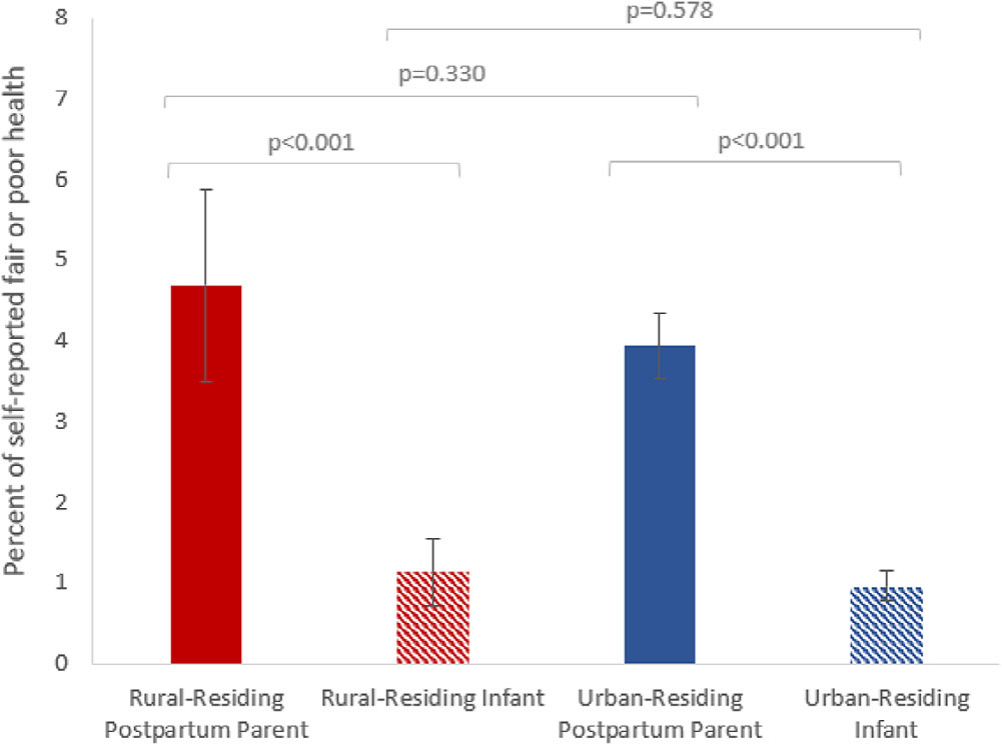
Self‐rated health status of postpartum parents and infants by rural–urban residence. This figure is a bar chart showing the percent of self‐rated fair or poor health between rural‐residing postpartum parents, rural residing infants, urban‐residing parents, and urban‐residing infants. Data reflect the full population, unweighted ns: rural‐residing postpartum parent (*n* = 2019), rural‐residing infant (*n* = 2191), urban‐residing postpartum parent (*n* = 12,112), urban‐residing infant (*n* = 13,088). Data are weighted to account for complex survey design, nonresponse bias, and multiple survey cycles. Within postpartum parent–infant differences account for household correlation.

Self‐rated fair or poor health was statistically similar among rural and urban postpartum parents and infants (Figure 1). Within both rural and urban dyads, more postpartum parents rated their health as fair or poor compared to fair or poor health among their infants (*p* < 0.001).

### Health care utilization: rural–urban differences

There were differences in where rural postpartum parents and infants received typical care. Compared to urban dyads, a lower proportion of rural dyads were seen in doctor's offices and a higher proportion were seen in clinics/health centers and the hospital ED (Table 2). Compared to urban postpartum parents, those in rural areas were less likely to see an obstetrician–gynecologist (*p* = 0.002), had a higher number of ED visits in the last year (*p* = 0.030), and reported more hospitalizations (*p* = 0.041) (Table 2).

**TABLE 2:** Health care utilization among postpartum parent and infants in rural and urban areas.

	Rural				Urban				Rural vs. urban parent	Rural vs. urban infant
	Postpartum parent	Infant	Parent vs. infant^a^		Postpartum parent	Infant	Parent vs. infant^a^			
			Difference	*p* Value			Difference	*p* Value		
Sample population, unweighted *n*	930	1194			4977	7021				
**Type of place for usual medical care**				0.853				0.998	0.000	0.002
Clinic/health center	35.7 (30.3–41.2)	32.3 (27.7–36.9)	1.8 (–5.2, 8.7)		23.0 (21.5–24.5)	24.5 (23.1–25.8)	0.1 (–2.3, 2.4)			
Doctor's office	57.9 (52–63.9)	66.1 (61.6–70.7)	−1.5 (–7.5, 4.5)		73.1 (71.5–74.6)	73.7 (72.3–75.1)	−0.1 (–2.0, 1.9)			
Hospital emergency room	1.78 (0.68–2.88)	DS			1.6 (1.2–2.1)	DS				
Other/no one place/unknown	4.5 (2.0–7.1)	1.6 (0.6–2.7)	−0.3 (–1.3, 0.7)		2.3 (1.8–2.8)	1.8 (1.4–2.2)	−0.0 (–0.4, 0.4)			
**Total office visits in the last 12 months**				<0.001				<0.001	0.154	0.100
None	7.9 (5.4–10.3)	6.5 (3.9–9.0)	−7.0 (–9.6, –4.4)		6.3 (5.4–7.1)	4.3 (3.7–5.0)	−5.4 (–6.2, –4.6)			
1	5.4 (3.9–6.9)	8.6 (6.7–10.5)	−6.1 (–8.5, –3.7)		6.0 (5.2–6.9)	8.9 (8.0–9.8)	−6.7 (–7.7, –5.6)			
2–3	8.1 (5.9–10.2)	25.5 (21.8–29.2)	−10.7 (–15.0, –6.5)		11.3 (10.2–12.4)	28.5 (27.0–30.0)	−12.2 (–14.0, –10.4)			
4–5	7.7 (5.7–9.7)	26.6 (23.5–29.7)	−4.7 (–7.1, –2.4)		7.7 (6.8–8.6)	27.7 (26.3–29.1)	−4.6 (–5.5, –3.6)			
6+ visits	69.7 (66.1–73.3)	31.0 (27.2–34.9)	27.7 (19.3, 36.1)		67.4 (65.6–69.2)	29.5 (28.1–30.9)	28.0 (24.8, 31.2)			
Unknown	1.3 (0.6–2.1)	1.8 (0.6–3.1)	0.8 (0.2, 1.5)		1.3 (0.9–1.7)	1.0 (0.8–1.3)	0.8 (0.5, 1.1)			
**Saw a specific clinician or had a specific visit in the past 12 months**										
OBGYN	82.7 (79.4–86.0)	NA	–	–	88.0 (86.9–89.0)	NA	–	–	0.002	–
NP/PA/midwife	39.3 (35.3–43.3)	NA	–	–	39.5 (37.7–41.3)	NA	–	–	0.790	–
Well child check	NA	87.9 (84.9–90.8)	–	–	NA	88.7 (87.6–89.7)	–	–	–	0.673
**Number of ED visits in the last year**				<0.001				<0.001	0.030	0.407
None	55.8 (51.4–60.2)	75.3 (71.6–78.9)	−22.5 (–29.5, –15.4)		60.7 (58.9–62.4)	78.2 (76.9–79.4)	−20.9 (–24.2, –17.7)			
1	21.0 (17.9–24.2)	16.1 (13–19.2)	8.7 (5.9, 11.5)		21.9 (20.5–23.4)	14.1 (13–15.1)	10.3 (8.6, 11.9)			
2–3	15.1 (12.2–18)	6.4 (4.6–8.1)	9.6 (6.0, 13.3)		11.9 (10.7–13.1)	5.8 (5.1–6.6)	7.1 (5.7, 8.4)			
>3	7.1 (4.2–9.9)	2.0 (1.0–3.0)	3.5 (1.8, 5.3)		4.6 (3.9–5.3)	1.5 (1.1–1.8)	3.1 (2.4, 3.9)			
Unknown	1.1 (0.4–1.8)	–			1.0 (0.6–1.3)	–				–
Full population, unweighted *n*	2019	2191			12,112	13,088				
**Number of hospitalizations in the last year** ^b^				<0.001				<0.001	0.041	0.019
None	90.0 (88.3–91.7)	93.9 (92.7–95.0)	−4.6 (–6.8, –2.5)		95.2 (94.8–95.6)	92.2 (91.6–92.7)	−4.0 (–4.8, –3.2)			
1	6.6 (5.2–8.1)	4.8 (3.7–6.0)	3.2 (1.7, 4.7)		3.5 (3.1–3.9)	5.3 (4.8–5.7)	2.7 (2.1, 3.2)			
2+	3.3 (2.4–4.2)	1.2 (0.9–1.6)	1.4 (0.7, 2.1)		1.1 (0.9–1.4)	2.4 (2.1–2.8)	1.2 (0.9, 1.5)			

*Note*: Data are weighted to account for complex survey design, nonresponse bias, and multiple survey cycles.

Abbreviations: DS, did not specify (absence of item specific data points); OBGYN, obstetrician–gynecologist; NP, nurse practitioner; PA, physician assistant; ED, emergency department; NA, not applicable.

^a^ Predicted postpartum parent–infant differences account for household correlation, and thus sometimes diverge from the weighted percents presented separately in the postpartum parent and infant columns.

^b^ The number of hospitalizations in the last year excludes the birth hospitalization.

### Health care utilization: postpartum parent–infant differences

Within the postpartum parent–infant dyad, differences in office visit patterns for both rural and urban postpartum parents were bimodal. A subset of postpartum parents were less likely to have an office visit compared to their infants (7.0 percentage points [pp] and 5.4 pp lower prevalence among rural and urban postpartum parents, respectively). While another subset had ≥6 visits at a rate that was 27.7 pp and 28.0 pp higher among rural and urban postpartum parents, respectively, than their infants (Table 2).

Postpartum parents visited the ED significantly more often than infants (e.g., urban postpartum parents had a 10.3 pp higher prevalence of having 1 ED visit compared to their infants). Compared to their infants, a higher proportion of postpartum parents were hospitalized (e.g., rural postpartum parents had a 3.2 pp higher prevalence of having 1 hospitalization compared with their infants) (Table 2).

### Barriers to accessing health care: rural–urban differences

Disruptions in usual medical care, periods of time without health insurance coverage, and losing Medicaid coverage after pregnancy were significantly more frequent among postpartum parents in rural areas than those in urban areas (Table 3). Significantly more rural postpartum parents experienced delays in medical care in the past year compared to those in urban areas (20.3% vs. 15.8%, *p* = 0.009), which was not statistically different between rural and urban infants (8.6% vs. 8.3%, *p* = 0.768) (Table 3).

**TABLE 3:** Barriers to health care among postpartum parents and infants in rural and urban areas.

	Rural				Urban					
	Postpartum parent	Infant	Parent vs. infant^a^		Postpartum parent	Infant	Parent vs. infant^a^		Rural vs. urban parent	Rural vs. urban infant
			Difference	*p* Value			Difference	*p* Value		
Full population, unweighted *n*	2019	2191			12,112	13,088				
Had no health coverage at some point during past 12 months	7.6 (6.2–9.0)	2.3 (1.6–3.0)	6.4 (4.6, 8.2)	<0.001	5.9 (5.4–6.5)	3.0 (2.6–3.4)	3.7 (3.0, 4.5)	<0.001	0.023	0.129
Medicaid/medical coverage stopped after pregnancy	10.0 (7.9–12.0)	NA	NA		7.2 (6.7–7.8)	NA	NA		0.006	NA
Changed usual place for health care for insurance reasons	1.2 (0.6–1.8)	1.33 (0.29–2.37)	−0.1 (–1.2, 1.0)	0.857	2.8 (2.2–3.4)	1.3 (1.0–1.7)	1.5 (0.8, 2.2)	<0.001	0.002	0.978
Sample population, unweighted *n*	930	1194			4977	7021				
**Delayed medical care in the past 12 months**										
Any reason	20.3 (16.9–23.7)	8.6 (6.7–10.5)	13.5 (9.7, 17.2)	<0.001	15.8 (14.6–17.1)	8.3 (7.5–9.2)	8.9 (7.3, 10.5)	<0.001	0.009	0.768
Cost	7.6 (6.3–8.9)	1.5 (0.8–2.1)	7.8 (6.0, 9.6)	<0.001	6.4 (5.9–6.9)	1.6 (1.3–1.9)	6.1 (5.3, 6.9)	<0.001	0.101	0.513
Couldn't get appointment soon	6.2 (4.4–7.9)	4.5 (3.0–6.0)	2.2 (–1.9, 6.3)	0.293	5.6 (4.7–6.4)	3.0 (2.5–3.5)	2.7 (1.3, 4.0)	<0.001	0.198	0.073
Doctor's office not open	4.0 (2.2–5.9)	1.8 (0.8–2.8)	2.4 (–0.7, 5.5)	0.127	2.7 (2.1–3.3)	1.6 (1.2–1.9)	0.9 (–0.0, 1.9)	0.059	0.081	0.663
Couldn't get through by phone	3.6 (1.7–5.4)	1.1 (0.4–1.9)	2.7 (–0.4, 5.8)	0.089	3.2 (2.6–3.8)	1.4 (1.1–1.7)	1.4 (0.4, 2.5)	0.008	0.319	0.625
Lacked transportation	2.5 (1.4–3.6)	2.6 (1.4–3.8)	1.0 (–1.3, 3.3)	0.394	2.2 (1.8–2.7)	1.8 (1.4–2.2)	0.3 (–0.6, 1.2)	0.490	0.194	0.274
Wait too long in doctor's office	5.03 (3.0–7.1)	3.3 (2.1–4.6)	1.6 (–1.6, 4.9)	0.326	4.7 (3.9–5.5)	2.7 (2.2–3.1)	1.9 (0.6, 3.2)	0.004	0.328	0.396

*Note*: Data are weighted to account for complex survey design, nonresponse bias, and multiple survey cycles.

Abbreviation: NA, not applicable.

^a^ Predicted postpartum parent–infant differences account for household correlation, and thus sometimes diverge from the weighted percents presented separately in the postpartum parent and infant columns.

### Barriers to accessing health care: postpartum parent–infant differences

Within the dyad, postpartum parents were significantly more likely to experience uninsurance compared to their infants (6.4 pp and 3.7 pp more among rural and urban postpartum parents, respectively) (Table 3). The proportion of postpartum parents reporting delayed medical care was 13.5 pp higher than infants within rural dyads and 8.9 pp higher within urban dyads. Cost was a more prevalent reason for delayed care for postpartum parents than infants within the same household, regardless of residence (Table 3). Inability to get an appointment or get through on the phone was experienced by a higher proportion of urban postpartum parents than infants (Table 3).

## DISCUSSION

For postpartum parent–infant dyads in the United States, health care access and utilization differed between rural and urban populations, particularly among postpartum parents. Health, health care use (aside from hospitalizations), and barriers to accessing care were generally similar between rural and urban infants. However, we found substantial differences within dyads. These within dyad differences have not been closely examined and may speak to differential access to care^24^ or reflect differing perceptions and prioritization of infant care over that of the postpartum parent.^39^, ^40^ We focused on the experiences reported by postpartum parents, which highlight the need to improve access to care for the dyad potentially through better integration of essential services.

### Postpartum parent rural–urban differences in health care utilization

These findings demonstrate that rural postpartum parents have less access to specialized clinical care (obstetrics‐gynecology) and more concerning health care utilization patterns (ED visits and hospitalizations), compared with urban postpartum parents. While there was no significant difference in the number of office visits reported, there was a difference in obstetrician–gynecologist visits. This likely reflects limited availability of these types of clinicians in rural areas, given that 40% of rural counties lack an obstetrician–gynecologist.^41^ Limited availability of specialized obstetric care in rural areas may result in increased ED utilization and postpartum hospitalizations, as was seen in this study. The comparatively limited access to specialty services in rural areas could be one reason childbirth in an urban hospital has been associated with fewer postpartum ED visits.^42^ Furthermore, prior studies show rural residents have a higher burden of pre‐pregnancy comorbidities, serious adverse childbirth events, and worse postpartum health outcomes.^3^, ^5^, ^43^, ^44^, ^45^, ^46^ These disparities in health, pregnancy, and childbirth‐related complications, may contribute to the rural–urban differences in postpartum acute care utilization and hospitalization seen in this study. Decisions to seek care in an ED instead of an outpatient clinic may be compounded by loss of insurance coverage, which was more frequent among rural postpartum parents.^47^ Additionally, rural postpartum parents reported significantly more barriers that delayed care, which could contribute to worsening clinical conditions and result in acute care utilization. These findings contribute to a growing body of evidence for structural urbanism, indicating how lack of access to obstetric‐specific care around childbirth, disruptions in insurance coverage, and increased reported barriers to care continue to disadvantage rural postpartum parents.^8^, ^30^, ^31^, ^48^

### Infant rural–urban differences in health care utilization

While differences in health care utilization and barriers to accessing care were less frequent between rural and urban infants, notable differences existed in the location of routine care and the number of hospitalizations they experienced. Specifically, a lower proportion of rural infants were hospitalized, which is consistent with prior research.^49^ This could reflect a lower need among rural children; however, other trends suggest it may indicate unmet need. Concurrent with this study's time period (2006–2018), data from 2008–2018 found a marked decrease in the number of rural inpatient pediatric units and data from 2009–2019 report a 4‐fold decrease in pediatric hospitalizations at rural hospitals.^50^, ^51^ Collectively, these data reflect ongoing regionalization of inpatient pediatric care through referral and transfer, and decreasing availability of pediatric inpatient services in rural communities.^52^ The combination of less pediatric bed availability and shifts in clinical thresholds for inpatient pediatric admissions (based on the rising proportion of pediatric admissions with chronic disease diagnoses) may also contribute to rural–urban differences.^51^ In addition, the number of clinicians comfortable with infant care is declining in rural areas, though our analyses did not reveal rural–urban differences in visit frequency.^9^

### Postpartum parent–infant differences in health, health care utilization, and barriers to accessing health care

One of the most consistent findings across both rural and urban settings was the within‐dyad differences between postpartum parents and infants. Infants generally had better overall self‐rated health reported, less emergent and hospital‐based health care utilization, and fewer barriers to care compared to postpartum parents. They also experienced fewer disruptions in health care coverage than their parents. This differential access to health care coverage may be detrimental to dyads, especially given that 10% of households reported there was only one adult in the home. In order to care for their infants it is important for parents and caregivers to be physically, mentally, and financially well. Yet, this analysis shows that US health care systems are not consistently able to facilitate access to recommended care for the postpartum parent–infant dyad. The differences in barriers (especially cost) within dyads illustrates that a higher value may be placed on infant health than postpartum parent health. The prioritization of an infant's health over one's own is a common parenting phenomenon; however, it has potential downstream consequences on the health of the postpartum parent and the dyad as a whole.^39^, ^40^

### Implications for practice and/or policy

These findings reveal opportunities to better optimize health and health care delivery for both postpartum parents and infants; such investments are an opportunity to improve overall dyad health and well‐being. Improved care could be achieved through family medicine models that serve both adults and infants throughout the perinatal period or through alignment and integration between obstetric and pediatric sites and clinicians. Federal and state policy efforts to improve access to maternity care, especially in rural areas, should include family medicine as an essential component.^53^, ^54^, ^55^ The number of family medicine practices that provide pregnancy‐related care is low and falling, so investments may be required to maintain this essential resource.^56^, ^57^ Given findings that infant health is prioritized relative to parent health within rural and urban dyads, there are also opportunities to leverage interactions with postpartum parents in pediatric settings. Postpartum parents are present at 93% of pediatric visits in the first two years of life.^24^, ^58^ Experience with screening for postpartum depression in pediatric settings provides one example of how basic maternal services could be incorporated into pediatric settings.^59^ Yet, it can be challenging to provide interventions to postpartum parents who are not often the patients of pediatric clinicians.^59^ On successful example, is depression screening that has been supported by innovative payment models allowing pediatric sites to bill for this care to postpartum parents. Other services may require enhanced referral and consultation pathways between pediatric and obstetrical care providers, as seen in recent pilots of postpartum hypertension screening in infant care settings.^60^

Changes to insurance status in the perinatal period can be a barrier to health care access in the year after childbirth.^61^ This study includes data from 2006 to 2018. During this period, changes in state and federal policies were associated with increased insurance coverage for rural residents and low‐income women in some states.^61^, ^62^ However, most state‐level policy changes did not specifically change pregnancy‐related Medicaid eligibility thresholds and perinatal uninsurance remained higher among rural residents, compared to their urban counterparts.^31^, ^61^, ^63^ Additionally, changes related to state Medicaid expansion decisions did not mitigate rural–urban disparities in cost‐related barriers, access to routine care, or doctor visits among postpartum patients generally.^62^ Since 2021, 47 states have extended pregnancy‐related Medicaid coverage for 12 months postpartum. Future analysis of the NHIS data after the 2019 re‐design may therefore reflect the more recent policy context where insurance status changes in the year after birth are less common among low‐income women.^24^

### Limitations and strengths

This study has limitations. Rurality is a continuum and both rural and urban areas are diverse. Due to limitations of these data we used a dichotomous measure based on whether the county of residence was metropolitan (urban) or non‐metropolitan (rural), consistent with prior research.^31^, ^64^ While statistical analyses within the dyad accounted for correlation of outcomes within the same household, weighting to represent the US population of dyads specifically was not available for this analysis. Adult–child pair weights that incorporate the sampling probabilities of both adults and children and adjusted for pair‐level nonresponse were not developed and available in the NHIS data until the 2019 redesign.^38^ For these analyses, we instead used individual weights for pair‐level statistical analysis, which may bias results. However, if the number of infants across households is the same as postpartum parents, it is likely that pair weights and individual weights would be highly correlated, resulting in the same mean estimates.^38^ In our sample population of postpartum parents and infants, 95.5% were from the same household. In this cross‐sectional survey data, the age of the infant is not specified (e.g., 2 months vs. 10 months), which this may influence the number of visits reported for either member of the dyad (multiple recommended visits in the third trimester and in the first 6 months of infancy). This limits the ability to comment on the completeness of perinatal, postpartum, or early infant care. However, if respondents are distributed across the postpartum year, it is unlikely to bias results between rural and urban dyads. Similarly, although data were collected from postpartum parents and the hospitalization for childbirth is excluded in this analysis, we cannot distinguish if the reported health care utilization and barriers reflect portions of the prenatal period.

## CONCLUSIONS

Rural postpartum parents experience more challenges than their urban counterparts accessing postpartum health care, a pattern that is consistent with inequities faced by rural residents across the life course. Investments in rural health care infrastructure may support rural families. Additionally, within dyads in both rural and urban communities, postpartum parents prioritized health care access for their infants over themselves. Integrating and incentivizing care for postpartum parents alongside their infants may address differential use and access to care in this critical period.

## CONFLICT OF INTEREST STATEMENT

The authors have no conflicts of interest to disclose. The NIH had no role in the design and conduct of the study; in the collection, analysis, and interpretation of the data; and in the preparation, editing, or censuring of the manuscript.
